# Optimization of tamoxifen solubility in carbon dioxide supercritical fluid and investigating other molecular targets using advanced artificial intelligence models

**DOI:** 10.1038/s41598-022-25562-y

**Published:** 2023-01-24

**Authors:** Saad M. Alshahrani, Abdullah S. Alshetaili, Munerah M. Alfadhel, Amany Belal, Mohammad A. S. Abourehab, Ahmed Al Saqr, Bjad K. Almutairy, Kumar Venkatesan, Amal M. Alsubaiyel, Mahboubeh Pishnamazi

**Affiliations:** 1 Department of Pharmaceutics, College of Pharmacy, Prince Sattam Bin Abdulaziz University, P.O. Box 173, Al-Kharj, 11942 Saudi Arabia; 2 Department of Pharmaceutical Chemistry, College of Pharmacy, Taif University, Taif, 21944 Saudi Arabia; 3 Medicinal Chemistry Department, Faculty of Pharmacy, Beni-Suef University, Beni-Suef, 62514 Egypt; 4 Department of Pharmaceutics, College of Pharmacy, Umm Al-Qura University, Mecca, 21955 Saudi Arabia; 5 Department of Pharmaceutics and Industrial Pharmacy, Faculty of Pharmacy, Minia University, Minia, 61519 Egypt; 6 Department of Pharmaceutical Chemistry, College of Pharmacy, King Khalid University, Abha, 62529 Saudi Arabia; 7 Department of Pharmaceutics, College of Pharmacy, Qassim University, Buraidah, 52571 Saudi Arabia; 8 Institute of Research and Development, Duy Tan University, Da Nang, 550000 Viet Nam; 9 The Faculty of Pharmacy, Duy Tan University, Da Nang, 550000 Viet Nam

**Keywords:** Chemistry, Green chemistry

## Abstract

Particle size, shape and morphology can be considered as the most significant functional parameters, their effects on increasing the performance of oral solid dosage formulation are indisputable. Supercritical Carbon dioxide fluid (SCCO_2_) technology is an effective approach to control the above-mentioned parameters in oral solid dosage formulation. In this study, drug solubility measuring is investigated based on artificial intelligence model using carbon dioxide as a common supercritical solvent, at different pressure and temperature, 120–400 bar, 308–338 K. The results indicate that pressure has a strong effect on drug solubility. In this investigation, Decision Tree (DT), Adaptive Boosted Decision Trees (ADA-DT), and Nu-SVR regression models are used for the first time as a novel model on the available data, which have two inputs, including pressure, X1 = P(bar) and temperature, X2 = T(K). Also, output is Y = solubility. With an R-squared score, DT, ADA-DT, and Nu-SVR showed results of 0.836, 0.921, and 0.813. Also, in terms of MAE, they showed error rates of 4.30E−06, 1.95E−06, and 3.45E−06. Another metric is RMSE, in which DT, ADA-DT, and Nu-SVR showed error rates of 4.96E−06, 2.34E−06, and 5.26E−06, respectively. Due to the analysis outputs, ADA-DT selected as the best and novel model and the find optimal outputs can be shown via vector: (x1 = 309, x2 = 317.39, Y1 = 7.03e−05).

## Introduction

In current decades, numerous endeavors have been made to develop new therapeutic medicines and optimize the application of existing drugs^1–5^. One of the most important restrictions towards the development of therapeutic drugs is low drug bioavailability, which is mainly owing to insufficient drug solubilities and low dissolution rate^6^. Therefore, finding promising techniques to enhance and optimize the solubility of drugs is an important method. True recognition of the drug solubility is known as a major necessity for developing the supercritical technology in pharmaceutical processing.

Recently, the use of SCCO_2_ fluid has been recently of paramount interest in pharmaceutical industry for dissolution of various types of drugs and subsequent nanonization^7–9^. The presence of different advantages such as ease of operation, eco-friendliness, and the non-existence of organic solvents in the production process has increased the interest of scientists to use SCCO_2_ fluid for enhancing the solubility of drugs. The measurement of drug solubility is known as an important key point towards the development of the supercritical technology. If a specific drug possesses enough solubility in the solvent, its process can be feasible via the supercritical technology^9–11^.

Over the last fifteen years, development of mathematical modeling through artificial intelligence (AI) and machine learning (ML) approaches have found its undeniable role on various research and development (R&D)/industrial investigations such as membrane separation, pharmaceutics, chemical reactors, nanotechnology and so on. Owing to significant cost of experimental investigation of drugs solubility in laboratory, ML techniques have paved the way to predict drugs solubility because of their brilliant advantages such as automated nature and predictive ability.^12–15^.

Machine Learning (ML) is the most popular discipline for modelling data, and it may be regarded as the cornerstone of the subject of Data Science (DS). Supervised ML utilizes many approaches like regression trees, vector machines, and neural networks to train the computer. This model plays multiple applications in various scientific fields, mainly were challenging and costly experiments are performed. This branch of artificial intelligence predicts and models future data based on existing data^16–18^. Decision Trees are one of the most popular ML models. The central premise of a decision tree is to divide a complex problem into numerous more straightforward problems, which may result in a solution that is easier to grasp. Data features are predictor variables in a decision tree methodology, whereas the class to be mapped is the target variable^19^.

Boosting is a common and essential strategy in ensemble learning called enhanced learning. By integrating the essential predictors, boosting enhances prediction outcomes. AdaBoost is a popular Boosting technique can add numerous base learners to provide more better estimations^20–22^. NU-SVR is another base predictor. Epsilon-SVR and NU-SVR are distinguished by the way the training problem is parametrized. Both cost functions incorporate a form of hinge loss. The nu parameter in NU-SVR allows for control over the quantity of support vectors included in the resultant model. The exact identical problem can be solved with the necessary parameters.

In this investigation, Decision Tree (DT), Adaptive Boosted Decision Trees (ADA-DT), and Nu-SVR regression models are utilized for the first time as a novel model on the available data. With an R-squared score, DT, ADA-DT, and Nu-SVR showed results of 0.836, 0.921, and 0.813, respectively. Also, in terms of MAE, they showed error rates of 4.30E−06, 1.95E−06, and 3.45E−06. Another metric is RMSE, in which DT, ADA-DT, and Nu-SVR showed error rates of 4.96E−06, 2.34E−06, and 5.26E−06, respectively. Through the analysis outputs, ADA-DT has been considered as more significant and novel model to develop and enhance the solubility of tamoxifen.

### Data set

In this study, we are working with a tiny dataset that includes two inputs comprising X1 = P(bar) and X2 = T(K) (K). Also, output is Y = solubility. The number of data are 32 points retrieved from^23^. Dataset has been demonstrated below in Table 1^24^.

**Table 1:** Data set.

No	X1 = P(bar)	X2 = T(K)	Y(solubility)
1	120	308	4 × 10^−06^
2	160	308	4.94 × 10^−06^
3	200	308	5.49 × 10^−06^
4	240	308	5.96 × 10^−06^
5	280	308	3.99 × 10^−06^
6	320	308	3.88 × 10^−06^
7	360	308	8.38 × 10^−06^
8	400	308	1.24 × 10^−05^
9	120	318	2.15 × 10^−06^
10	160	318	5.79 × 10^−06^
11	200	318	8.95 × 10^−06^
12	240	318	7.27 × 10^−06^
13	280	318	3.40 × 10^−06^
14	320	318	7.03 × 10^−05^
15	360	318	4.01 × 10^−06^
16	400	318	1.39 × 10^−05^
17	120	328	1.79 × 10^−06^
18	160	328	5.13 × 10^−06^
19	200	328	1.05 × 10^−06^
20	240	328	5.48 × 10^−05^
21	280	328	2.31 × 10^−05^
22	320	328	2.04 × 10^−05^
23	360	328	2.50 × 10^−05^
24	400	328	4.41 × 10^−05^
25	120	338	1.52 × 10^−06^
26	160	338	3.84 × 10^−06^
27	200	338	1.05 × 10^−05^
28	240	338	2.08 × 10^−05^
29	280	338	3.13 × 10^−05^
30	320	338	1.95 × 10^−05^
31	360	338	5.47 × 10^−05^
32	400	338	6.0 × 10^−05^

## Methodology

### Decision tree

**Figure 1:**
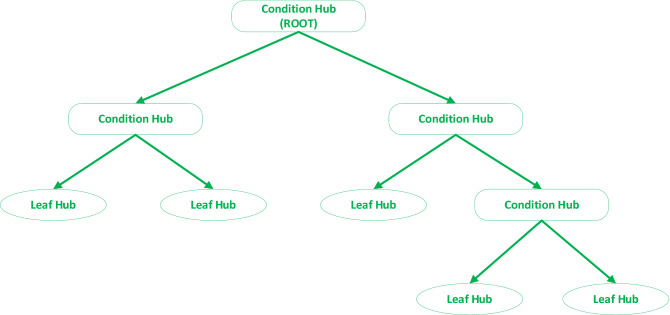
Decision tree sample architecture.

Trees are the significant data structures in various fields of artificial intelligence. A decision tree (DT) is a procedure commonly used to analyses data. A decision tree may handle either regression or classification tasks. A typical decision tree is made up of decision nodes (make a query on an input features), edges (result of a query and pass to the child node), and terminal or leaf nodes (generate the output)^25–27^, as shown in Fig. 1.

Each feature of a dataset is handled as a node or hub in the DT, via the root node to be unmatched. This approach will be more developed till a leaf node is identified. The decision tree's output can be the terminal node^19,28,29^. Some of the well-known decision tree induction algorithms such as CART^19^, CHAID^25^, C4.5, and C5.0^27,30^.

### AdaBoost

Freund and Schapire invented the AdaBoost^31^ to solve the binary classification problem. In AdaBoost method, the fundamental concept is to create several weak predictors sequentially using the training data subset and then merge them using a given technique. First, an equal-weighted training data is used to build the weak predictor. However, the weights of the examples in the training subset that were incorrectly estimated are raised. The new weighted training data is then used to build the weak predictor for the next round. After repeating the above technique, multiple weak predictors are obtained, and each predictor is assigned a score based on the related classification error. Using some rule to combine all weak predictors will result in a final strong predictor. Multiple AdaBoost variants have been implemented, each with its advantages and purposes^31–33^.

Each *x*_*i*_ instance’s weight *w*_*i*_ is set proportionally to the possibility of being accurately estimated, and implicitly proportionally to the predictor *T*_*t*_ error *t*. Furthermore, each predictor decision on a new example’s final prediction is weighted according to its performance during the learning^22,34,35^.

Following steps generally shows AdaBoost workflow:

- Begin with uniform sample weights.
- Initial number of predictors: *M*.
- For k in [1,…,M]:
  - Develop a base learner L_k_ via a weighted sample.
  - Test L_k_ on all data.
  - Set new weight for L_k_ using a weighted error.
  - Set weights for each sample data point.

This approach has several advantages, the most prominent of which is simpler to use and requires fewer hyper-parameters to be tuned. AdaBoost is not prone to overfitting because of its design and methodology^35^.

### Nu-SVR

A set of input and output parameters supplied as basic configuration *{(x*_*1*_*, y*_*1*_*), …, (x*_*n*_*, y*_*n*_*)}*. The goal of the Nu-SVR method is to compute the correlation indicated in the following Equation, as *f(x)* must in neighborhood of value of *y* as possible. It should also be as flat as feasible. Since we want to avoid over-fitted models in this investigation^36–38^.

1 $${f\left( x \right) = wT\;\Phi \left( x \right) + b}$$

In this equation, *Φ*(*x*) is declared as the non-linear function mapping the input space to space of higher dimensions and b denotes the bias. *w*^*T*^ is also stands for the weight vector. Optimization is the primary objective of the task: Closeness and flatness are two of the fundamental aims of this challenge, which is why the main goal is to optimize^37–41^:

2 $$\frac{1}{2}\left| {\left\lceil w \right\rceil } \right| + {\text{C}}\left\{ {Y \cdot\upvarepsilon + \frac{1}{n}\mathop \sum \limits_{i = 1}^{n} \left( {\xi + \xi^{*} } \right)} \right\}$$

According to the conditions:

3 $${\text{y}}_{{\text{i}}} - \left\langle {w^{T} \cdot \Phi \left( x \right)} \right\rangle - b \le\upvarepsilon + \xi_{i}^{*} ,$$

4 $$\left\langle {w^{T} \cdot \Phi \left( x \right)} \right\rangle + b - y_{i} \le\upvarepsilon + \xi_{i} ,$$

5 $$\xi_{i}^{*} ,\xi_{i} \ge 0$$

here ɛ is a distance between the f(x) and its actual amount. Also, *ξ, ξ*_*i*_ are extra slack variables depicted in^42^, declares that distance of value ξ above ɛ error are reasonable. The parameter *C*, define as the regularization amount, indicates an equilibrium on the tolerance of error ɛ and flatness of *f* ^38^.

So, *Y* (0 < *Y* < 1) shows the upper bound for the function of margin errors in training amounts and defines the lower bound for the fraction of support vectors. Furthermore, to address the first issue, the dual statement has been created through constructing the Lagrange function^38^:

6 $$\begin{aligned} & {\text{L}}:\frac{1}{2}\left| {\left\lceil w \right\rceil } \right|^{2} + {\text{C}}\left\{ {Y \cdot\upvarepsilon + \frac{1}{n}\mathop \sum \limits_{i = 1}^{n} \left( {\xi + \xi^{*} } \right)} \right\} - \frac{1}{n}\mathop \sum \limits_{i = 1}^{n} \left( {\eta \xi + \eta^{*} \xi^{*} } \right) \\ & \quad - \;\frac{1}{n}\mathop \sum \limits_{i = 1}^{n} \left( {\upvarepsilon + \xi_{i} + y_{i - } w^{T} \cdot \Phi \left( x \right) - b} \right) - \frac{1}{n}\mathop \sum \limits_{i = 1}^{n} \left( {\upvarepsilon + \xi_{i} + y_{i} + w^{T} \cdot \Phi \left( x \right) + b} \right) - \beta\upvarepsilon \\ \end{aligned}$$

*a*, a^*^, *η*, η^*^, *β* demonstrate the Lagrange multipliers, then *a*^(*)^ = *a*·a^*^, through maximize Lagrange function *W* = $\sum\nolimits_{i = 1}^{n} {\left( {a_{i} - a_{i}^{*} } \right) \cdot \Phi \left( x \right)}$ and leads to a problem with dual optimization^38^:

7 $${\text{Maximizes}} - \frac{1}{2}\mathop \sum \limits_{i = 1}^{n} \left( {a_{i} - a_{i}^{*} } \right) \cdot \left( {a_{j} a_{j}^{*} } \right) \cdot k\left( {x_{i} x_{j} } \right) + \mathop \sum \limits_{i = 1}^{n} y_{i} \left( {a_{i} - a_{i}^{*} } \right);$$

Subject to:

8 $$\mathop \sum \limits_{i = 1}^{n} \left( {a_{i} - a_{i}^{*} } \right) = 0 \left( 8 \right)$$

9 $$\mathop \sum \limits_{i = 1}^{n} \left( {a_{i} - a_{i}^{*} } \right) \le CY$$

10 $$a_{i} ,a_{i}^{*} \epsilon \left[ {0,\frac{C}{n}} \right].$$

Since *K*(*x*_*i*_,*x*_*j*_) stands for the kernel function defined through *K*(*x*_*i*_,*x*_*j*_) = *Φ*(*x*_*i*_)^*T*^·*Φ*(*x*_*j*_). The solution to recent Formula yields to the Lagrange multipliers a, a^*^. An estimate of the function *(L)* is obtained when weight *W* is swapped into recent equations:

11 $$f\left( x \right) = \mathop \sum \limits_{n = 1}^{n} \left( {a_{i} - a_{i}^{*} } \right) \cdot k(x_{i} ,x) + b$$

### Tamoxifen targets beside estrogen receptors

Pubchem web site was used for smiles retrieval of tamoxifen (https://pubchem.ncbi.nlm.nih.gov/compound/Tamoxifen#section=InChI). Smiles code obtained was as the following (CCC(=C(C1=CC=CC=C1)C2=CC=C(C=C2)OCCN(C)C)C3=CC=CC=C3), this code was fed into LigTMap web server (https://cbbio.online/LigTMap/) to search for other molecular targets of tamoxifen, selected target classes in this search are Anticogulant, Beta_secretase, Bromodomain, Carbonic_Anhydrase, Hydrolase, Isomerase, Kinase,Ligase, Peroxisome, Transferase, Diabetes, HCV, Hpyroli, HIV, Influenza and Tuberculosis. Also, this smile code was inserted in swissADME web server (http://www.swissadme.ch/index.php) to investigate its boiled egg model in addition to the physicochemical parameters.

## Results

After tuning of important hyper-parameters by run different combinations some metrics are needed to evaluate the accuracy of final models. The statistical measurements of RMSE, MAE, and R-squared is used to compare the accuracy of different models’ predictions^43,44^.

12 $${\text{RMSE}} = \sqrt {\frac{{\mathop \sum \nolimits_{j = 1}^{a} \left[ {z^{\prime } - z} \right]^{2} }}{a}}$$

13 $${\text{MAE}} = \frac{1}{a}\mathop \sum \limits_{j = 1}^{a} \left| {z^{\prime } - z} \right|$$

14 $$R^{2} = \frac{{a\sum z^{\prime } z - \sum z^{\prime } z}}{{\sqrt {\left[ {a\sum z^{\prime 2} - \left( {\sum z^{\prime } } \right)^{2} } \right]\left[ {a\sum z^{2} - \left( {\sum z} \right)^{2} } \right]} }}$$

The above three equations are used to calculate these metrics. Here z′ and z indicates the assessed and actual data, and *a* quantity of data.

**Figure 2:**
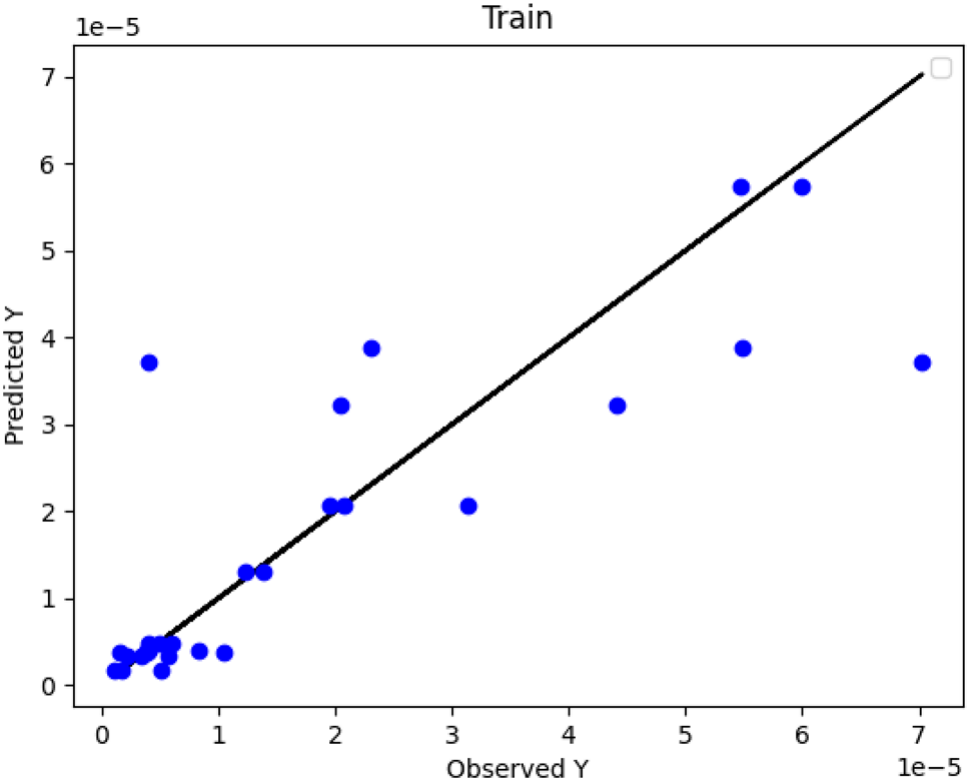
DT Model: actual versus predicted values/Y: solubility.

**Figure 3:**
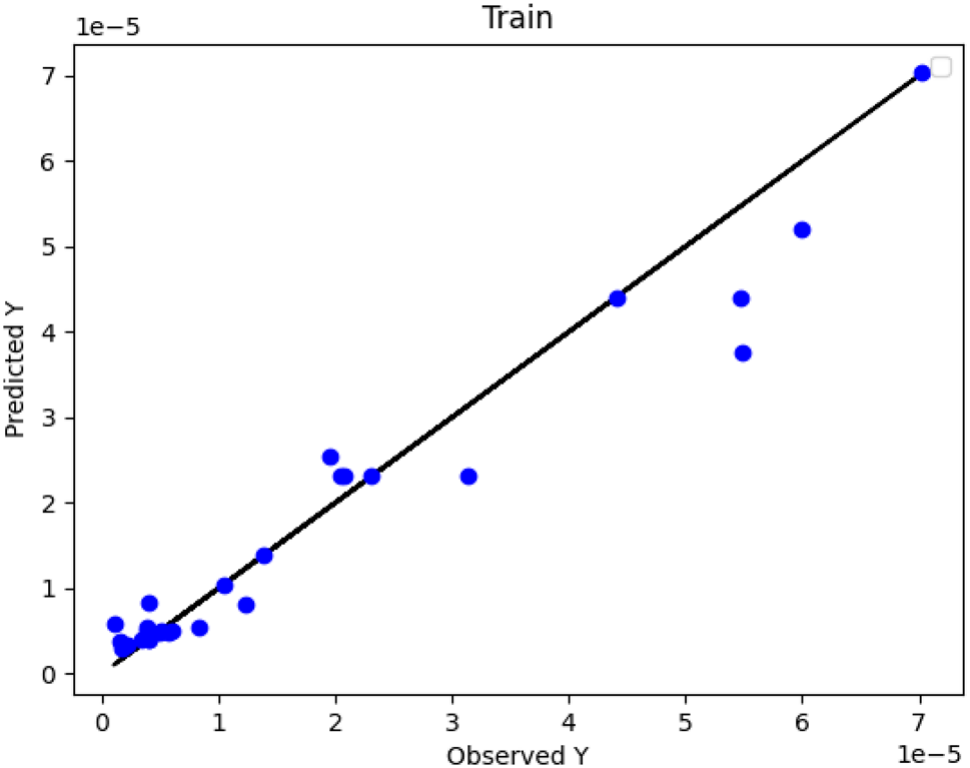
ADA-DT Model: actual versus predicted values/Y: solubility.

**Figure 4:**
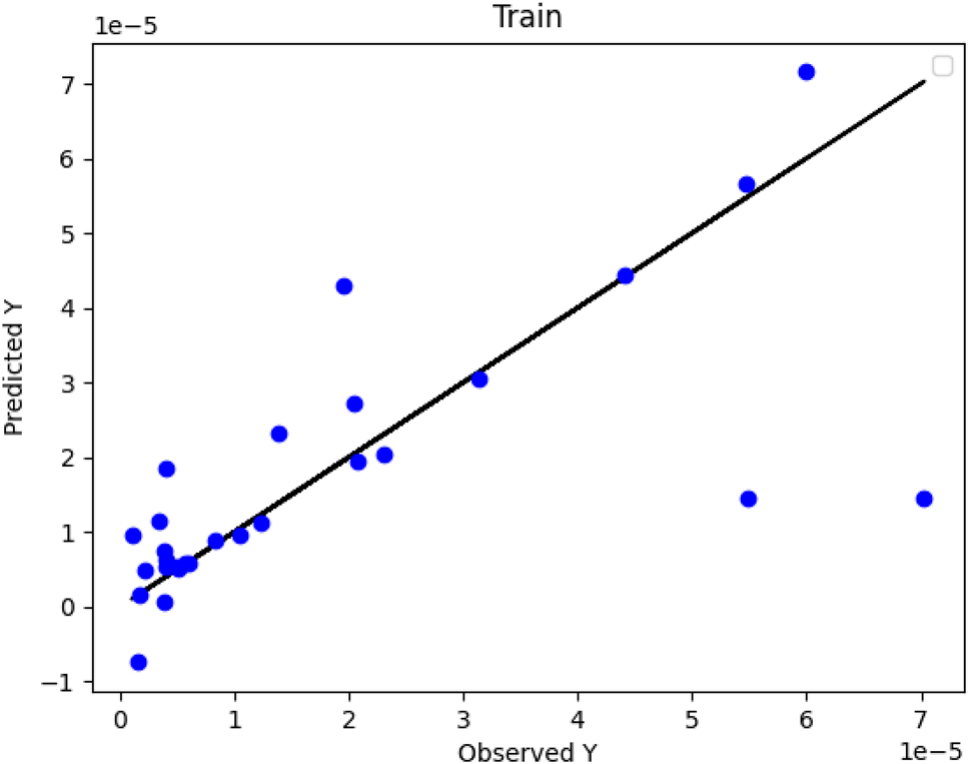
NU-SVR Model: actual versus predicted values/Y: solubility.

Figures 2, 3 and 4 numerically compare the real outcomes with estimated results in DT, ADA-DT and Nu-SVR machine-learning based models. As demonstrated, the ADA-DT model enjoys the greatest accuracy due to the presence of most points in a reasonable neighborhood of actual values. Considering the values of R^2^ and RMSE presented in Table 2, the ADA-DT is selected as an accurate model with the best generality.

**Table 2:** Outputs of different models.

Models	MAE	RMSE	R^2^
Decision tree (DT)	4.30E−06	4.96E−06	0.836
ADA-DT	1.95E−06	2.34E−06	0.921
Nu-SVR	3.45E−06	5.26E−06	0.813

**Figure 5:**
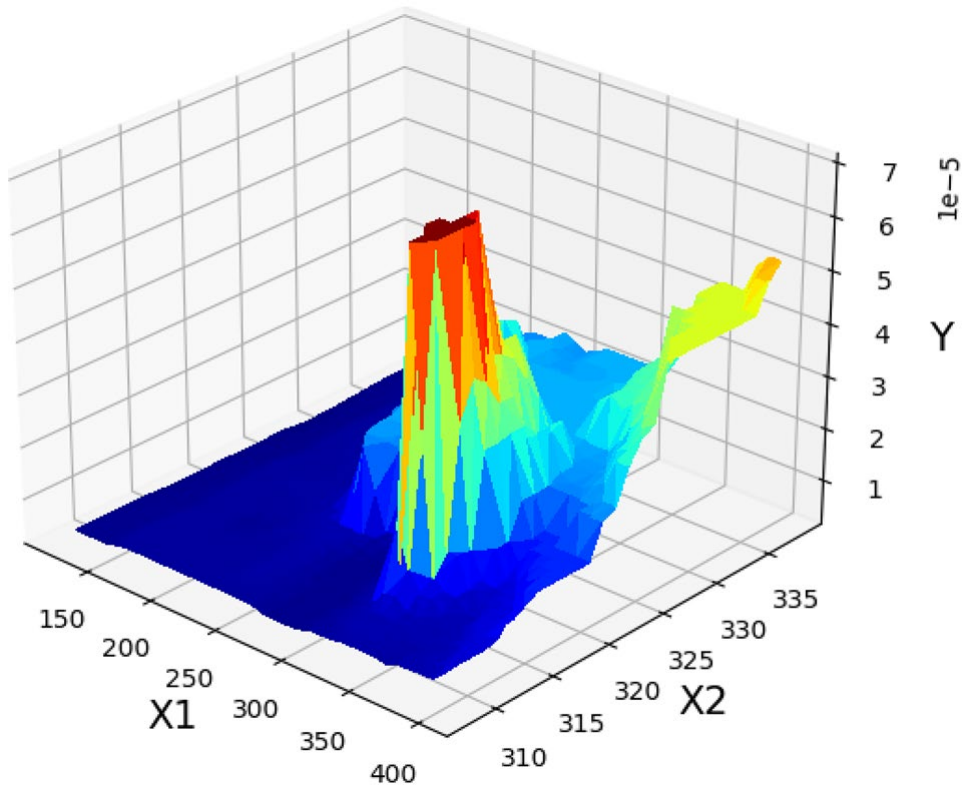
3D demonstrateion of inputs/outputs/Y: solubility/X_2_: temperature/X_1_: pressure.

**Figure 6:**
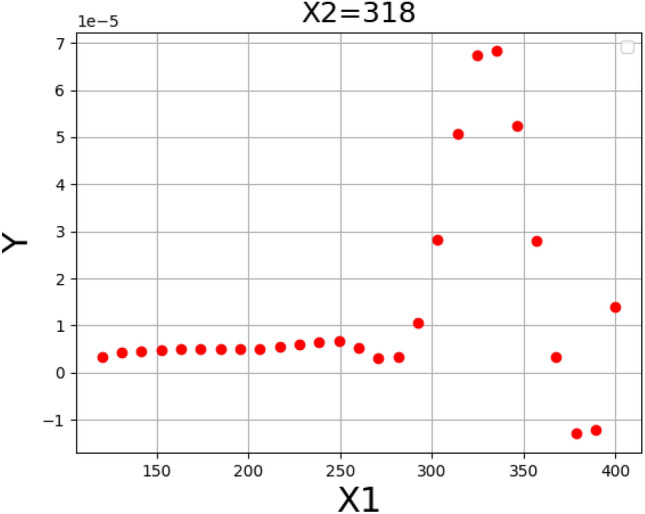
Solubility based on pressure/Y: solubility/X_1_: pressure.

**Figure 7:**
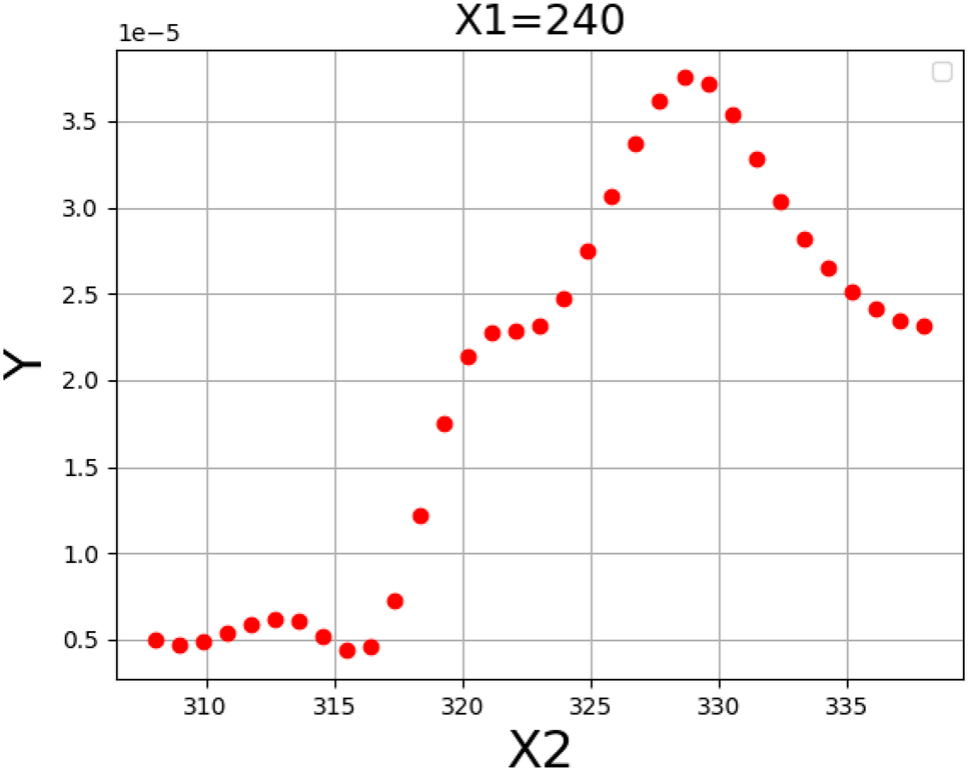
Solubility based on temperature/s: solubility/X_2_: temperature.

Figure 5 shows the result for assessing the influence of pressure and temperature as inputs on the solubility. Moreover, Figs. 6 and 7 illustrate two-dimensional depictions to individually analyzed the trends of two inputs on drug solubility^24^. Analysis of the figures implies the fact that increment of the pressure from 120 to 400 bar eventuates in a significant improvement in the solubility of tamoxifen. An enhancement of pressure significantly improves the amount of density accompanying with the solvating power, which positively enhances the solubility of drug in the SCCO_2_ system. About temperature, it must be said that the results show some complexities. In detail, the modeling outcomes show that the due to the existence of a threshold pressure, the influence of temperature show a reversal trend. In details, at the operational pressure lower than 240 bar, increasing the temperature decreases the solubility of tamoxifen because of a decrement in the density of solvent, with negative effect on the solvating power. According to the abovementioned analysis, it is proved that a shifting pressure named cross-over pressure is existed for the values less than this pressure (lower than 240 bar), the density reduction overcomes the sublimation pressure and therefore, the solubility of tamoxifen declines^23^. When the pressure goes beyond the cross-over pressure (240 bar), the role of pressure sublimation dominates the impact of density. Thus, by increasing the pressures at the pressures higher than cross-over pressure, the solubility of tamoxifen in the SCCO_2_ system increases^45,46^. The optimal values of pressure and temperature to obtain the highest amount of tamoxifen solubility is presented in Table 3^24^.

**Table 3:** Optimized pressure and temperature/ optimized solubility.

X1 = P(bar)	X2 = T(K)	Y(solubility)
309	317.39	7.03e−05

### Tamoxifen targets beside estrogen receptors and its boiled-egg model

**Figure 8:**
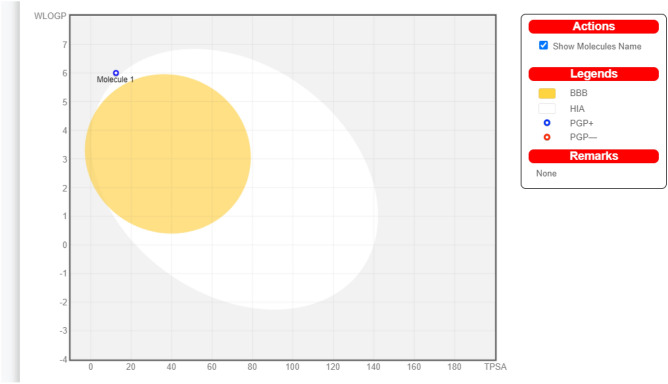
Tamoxifen boiled-egg model by SwissADME.

Tamoxifen continues to be used in treatment of estrogen positive breast cancer^47^. In the current research work we decided to investigate if there are other molecular targets for this crucial drug to figure a new way in its medicinal usage. We have used CADD techniques in our previous research work^48–51^ as they are useful tools in investigating diverse properties for different molecules. Through usage of SwissADME web server we could get the boiled-egg model of tamoxifen (Fig. 8 and supplementary data) that illustrates that tamoxifen with poor probability to penetrate BBB in addition to its poor GI-absorption. Additionally, the model showed that tamoxifen is PGP + which means that it can be effluated outside the cells by the action of P-glycoprotein. Being a substrate for P-glycoprotein increases the possibility of tamoxifen resistance. Improvement of tamoxifen solubility may lead to better physicochemical properties and better GI-absorbance.

**Figure 9:**
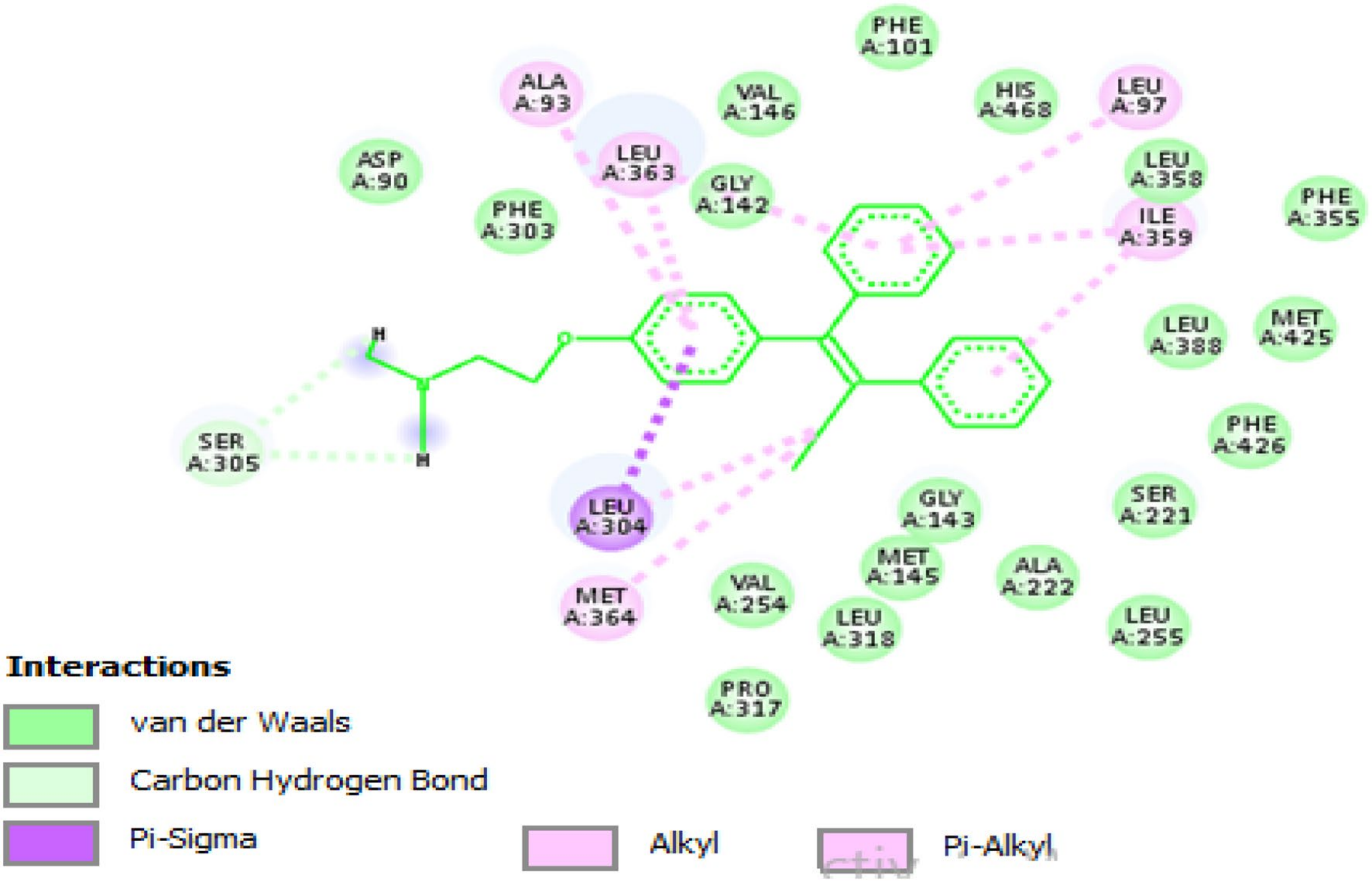
2D interactions of tamoxifen with CES1 protein binding site (pdb ID: 1ya4).

Furthermore, the other possible targets for tamoxifen were explored in this research work through LigTMap web server, all disease target classes were selected except estrogen. The obtained results revealed other seven putative tamoxifen targets other than estrogen (supplementary data files), these targets are divided into three Hydrolases (CES1 protein, bifunctional epoxide hydrolase 2 and LEUKOTRIENE A-4 HYDROLASE), two HCV (NON-STRUCTURAL PROTEIN 4A, SERINE PROTEASE NS3 and RNA-directed RNA polymerase), one predicted protein target for Beta_secretase (BETA-SECRETASE 1) and one protein target for Bromodomain (Bromodomain-containing protein 4). These plausible targets for tamoxifen are ranked according to LigTMap score as shown in Table 4, tamoxifen showed ligand similarity for these targets with range from 40 to 69%, the best ligand similarity score (0.689) was assigned for CTX ligand in CES1 protein (pdb Id: 1ya4). The results also revealed more than 55% binding similarity with Y80 lignad in Bromodomain-containing protein 4 (pdb ID: 4yh3). The best docking score was -7.925 kcal/mol with CES1 protein (pdb ID: 1ya4). Additionally, tamoxifen showed good docking score energy with these seven putative targets as shown in Table 4, docking score ranged from -5.759 to -7.925 kcal/mol. Figure 9 represents the 2D interactions of tamoxifen with CES1 protein binding site.

**Table 4:** Predicted putative tamoxifen targets retrieved from LigTMap.

Rank	Target class	Pdb ID	PSOVina2 docking score (kcal/mol)
1	Hydrolase	1ya4	− 7.925
2	Bromodomain	4yh3	− 6.148
3	HCV	4b6f	− 6.634
4	Hydrolase	4y2t	− 7.418
5	Hydrolase	5aen	− 7.729
6	Beta_secretase	1w51	− 5.759
7	HCV	3lkh	− 6.717

## Conclusion

In this research, to predict tamoxifen solubility, supercritical carbon dioxide is used as solvent. Experimental data have been provided through the literature, then analyzed to develop a predictive model. On the provided data, Decision Tree (DT), Adaptive Boosted Decision Trees (ADA-DT), and Nu-SVR regression models are employed through two parameters as inputs, Pressure and Temperature. Furthermore, solubility considered as output. DT, ADA-DT, and Nu-SVR demonstrate R-squared scores of 0.836, 0.921, and 0.813. The MAE error has been demonstrated by the rates of 4.30E−06, 1.95E−06, and 3.45E−06. RMSE as another statistic, revealed the error rates of 4.96E−06, 2.34E−06, and 5.26E−06 for DT, ADA-DT, and Nu-SVR, respectively. Based on these measurements and some visual inspection, ADA-DT has been considered as the best model to identify optimal values to predict drug solubility based on the optimized values x1 = 309, x2 = 317.39, Y1 = 7.03e−05). Furthermore, LigTMap web server has helped in identification of seven putative tamoxifen protein targets other than estrogen.

## Supplementary Information

Supplementary Information.

## Data Availability

All data are available within the published paper.
