# Author Correction: Optimization of tamoxifen solubility in carbon dioxide supercritical fluid and investigating other molecular targets using advanced artificial intelligence models

**DOI:** 10.1038/s41598-023-31514-x

**Published:** 2023-03-15

**Authors:** Saad M. Alshahrani, Abdullah S. Alshetaili, Munerah M. Alfadhel, Amany Belal, Mohammad A. S. Abourehab, Ahmed Al Saqr, Bjad K. Almutairy, Kumar Venkatesan, Amal M. Alsubaiyel, Mahboubeh Pishnamazi

**Affiliations:** 1 Department of Pharmaceutics, College of Pharmacy, Prince Sattam Bin Abdulaziz University, P.O. Box 173, Al-Kharj, 11942 Saudi Arabia; 2 Department of Pharmaceutical Chemistry, College of Pharmacy, Taif University, Taif, 21944 Saudi Arabia; 3 Medicinal Chemistry Department, Faculty of Pharmacy, Beni-Suef University, Beni-Suef, 62514 Egypt; 4 Department of Pharmaceutics, College of Pharmacy, Umm Al-Qura University, Mecca, 21955 Saudi Arabia; 5 Department of Pharmaceutics and Industrial Pharmacy, Faculty of Pharmacy, Minia University, Minia, 61519 Egypt; 6 Department of Pharmaceutical Chemistry, College of Pharmacy, King Khalid University, Abha, 62529 Saudi Arabia; 7 Department of Pharmaceutics, College of Pharmacy, Qassim University, Buraidah, 52571 Saudi Arabia; 8 Institute of Research and Development, Duy Tan University, Da Nang, 550000 Viet Nam; 9 The Faculty of Pharmacy, Duy Tan University, Da Nang, 550000 Viet Nam

Correction to: Scientific Reports 10.1038/s41598-022-25562-y, published online 24 January 2023

The original version of this Article contained an error in the Acknowledgements section, where the Grant Code of the Umm Al-Qura University was incorrect.

“The authors would like to thank the Deanship of Scientific Research at Umm Al-Qura University for supporting this work by Grant code: (22UQU4290565DSR93).”

now reads:

“The authors would like to thank the Deanship of Scientific Research at Umm Al-Qura University for supporting this work by Grant code: (23UQU4290565DSR060).”

The original Article has been corrected.

