# Supplementary material for: Optimization of tamoxifen solubility in carbon dioxide supercritical fluid and investigating other molecular targets using advanced artificial intelligence models
Source: Sci Rep. 2023 Jan 24;13:1313. doi: 10.1038/s41598-022-25562-y (PMC9873658; doi:10.1038/s41598-022-25562-y)
Supplement: Supplementary file 1 — Supplementary Information. [file 41598_2022_25562_MOESM1_ESM.pdf]

Result of Job ID #cfstz

Selected target classes: Anticogulant,Beta_secretase,Bromodomain,Carbonic_Anhydrase,Hydrolase,Isomerase,Kinase,Ligase,Peroxisome,Transferase,Diabetes,HCV,Hpyroli,HIV,Influenza,Tuberculosis
Description: Tamoxifen
Cutoff: 0.4

Input SMILES: CCC(=C(C1=CC=CC=C1)C2=CC=C(C=C2)OCCN(C)C)C3=CC=CC=C3

No. of targets found for selected target classes

Total	Hydrolase	HCV	Beta_secretase	Bromodomain
7	3	2	1	1

Target list

Rank	PDB	Target Class	Target Name	Ligand Name	Ligand Similarity Score	Binding Similarity Score	LigTMap Score	Predicted Affinity (-log M)	PSOVina2 Docking Score (kcal/mol)	PSOVina2 Docking Pose	Selec bind simil anal
1	1ya4	Hydrolase	CES1 protein	CTX	0.689	0.267	0.562	5.82	-7.925	3D PDB	Do
2	4yh3	Bromodomain	Bromodomain-containing protein 4	Y80	0.423	0.556	0.463	5.52	-6.148	3D PDB	Do
3	4b6f	HCV	NON-STRUCTURAL PROTEIN 4A, SERINE PROTEASE NS3	20L	0.404	0.5	0.433	5.383	-6.634	3D PDB	Do
4	4y2t	Hydrolase	Bifunctional epoxide hydrolase 2	49Q	0.405	0.091	0.311	5.82	-7.418	3D PDB	Do
5	5aen	Hydrolase	LEUKOTRIENE A-4 HYDROLASE	DP8	0.409	0.077	0.309	5.82	-7.729	3D PDB	Do
6	1w51	Beta_secretase	BETA-SECRETASE 1	L01	0.418	0	0.292	6.438	-5.759	3D PDB	Do

Rank	PDB	Target Class	Target Name	Ligand Name	Ligand Similarity Score	Binding Similarity Score	LigTMap Score	Predicted Affinity (-log M)	PSOVina2 Docking Score (kcal/mol)	PSOVina2 Docking Pose	Selec bind simil anal
7	3lkh	HCV	RNA-directed RNA polymerase	LT6	0.41	0	0.287	5.383	-6.717	3D PDB	Do

Copyright © 2022 University of Macau

0

In order to avoid security-related warning messages when switching to secured connection, you may want either to:

- confirm the exception on the next page, or
- import our CA key in your web browser

Click here to proceed.

SwissADME

This website allows you to compute physicochemical descriptors as well as to predict ADME parameters, pharmacokinetic properties, druglike nature and medicinal chemistry friendliness of one or multiple small molecules to support drug discovery.

The main article describing the web service and its underlying methodologies is SwissADME: a free web tool to evaluate pharmacokinetics, drug-likeness and medicinal chemistry friendliness of small molecules. *Sci. Rep.* (2017) 7:42717.
For details about development and validation of iLOG, please refer to this article: iLOGP: a simple, robust, and efficient description of *n*-octanol/water partition coefficient for drug design using the GB/SA approach. *J. Chem. Inf. Model.* (2014) 54(12):3284-3301.
For details about development and validation of the BOILED-Egg, please refer to this article: A BOILED-Egg to predict gastrointestinal absorption and brain penetration of small molecules. *ChemMedChem* (2016) 11(11):1117-1121.

Developed and maintained by the Molecular Modeling Group of the SIB | Swiss Institute of Bioinformatics.

Enter a list of SMILES here:

```
CCC(=C(C1=CC=CC=C1)C2=CC=C(C=C2)OCCN(C)C)C3=CC=CC=C3
```

Fill with an example | Clear | Run!

Hide BOILED-Egg

Retrieve data: POWERED BY ChemAxon

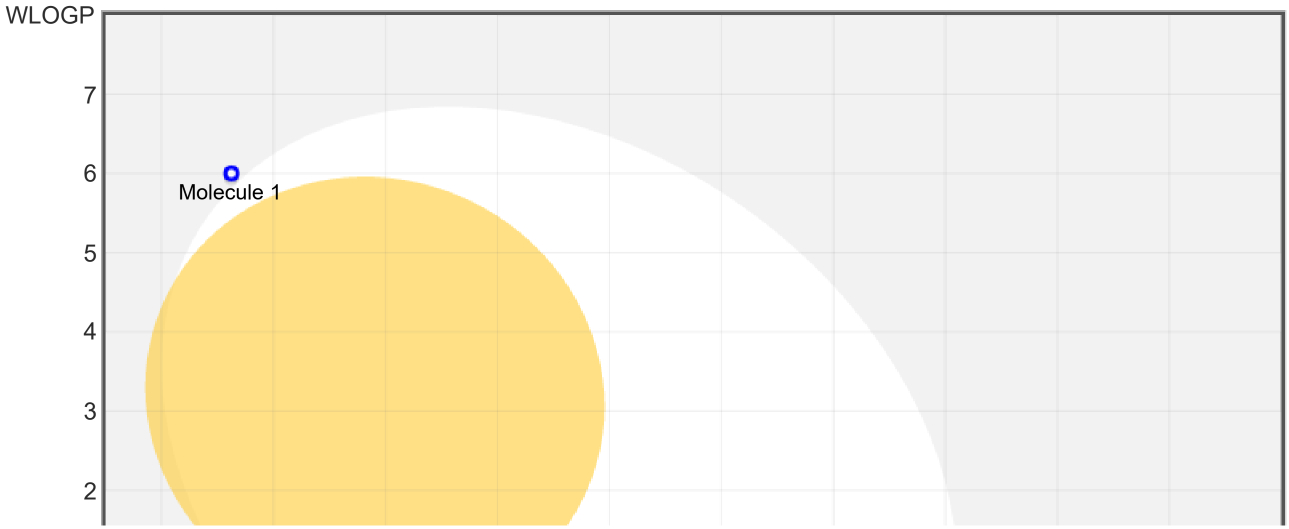

Actions

☑ Show Molecules Name

Legends

BBB

HIA

PGP+

PGP—

Remarks

None

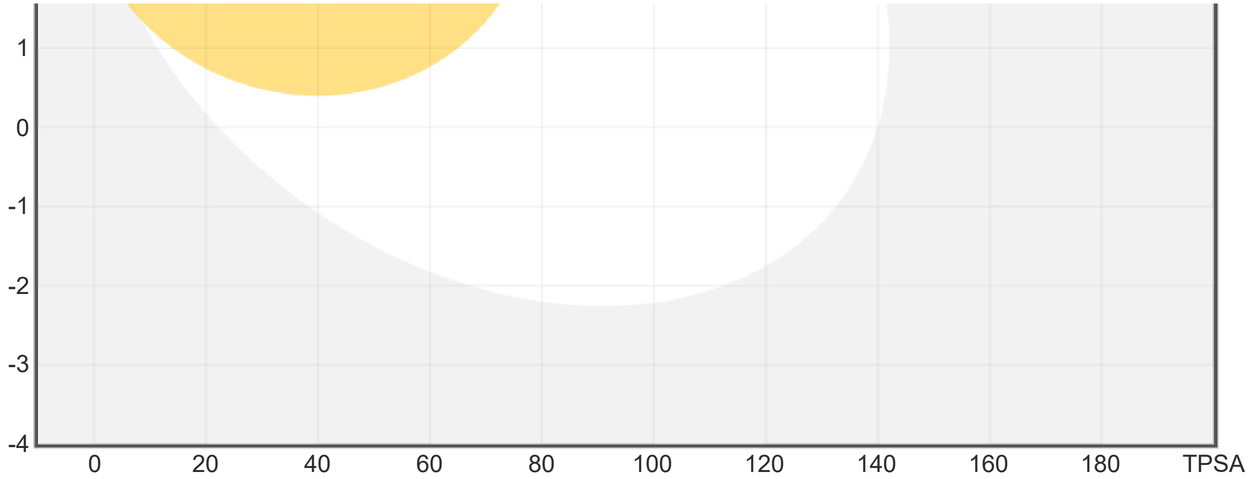

Molecule 1

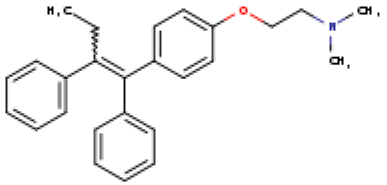

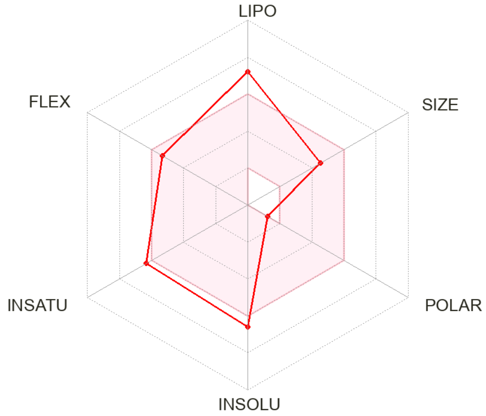

SMILES	CCC(=C(c1ccccc1)c1ccc(cc1)OCCN(C)C)c1ccccc1

Physicochemical Properties

Formula	C26H29NO
Molecular weight	371.51 g/mol
Num. heavy atoms	28
Num. arom. heavy atoms	18
Fraction Csp3	0.23
Num. rotatable bonds	8
Num. H-bond acceptors	2

Water Solubility

Log *S* (ESOL)	
**ESOL:** Topological method implemented from Delaney JS. 2004 J. Chem. Inf. Model.	-6.59
Solubility	9.56e-05 mg/ml ; 2.57e-07 mol/l
Class	
**Solubility class:** Log *S* scale Insoluble < -10 < Poorly < -6 < Moderately < -4 < Soluble < -2 Very < 0 < Highly	Poorly soluble
Log *S* (Ali)	-7.22
**Ali:** Topological method implemented from Ali J. et al. 2012 J. Chem. Inf. Model.	

Num. H-bond donors	0
Molar Refractivity	119.72
TPSA<br>**Topological Polar Surface Area:** Calculated from Ertl P. et al. 2000 J. Med. Chem.	12.47 Å²

Lipophilicity

Log $P_{o/w}$ (iLOGP)<br>**iLOGP:** in-house physics-based method implemented from Daina A et al. 2014 J. Chem. Inf. Model.	4.64
Log $P_{o/w}$ (XLOGP3)<br>**XLOGP3:** Atomistic and knowledge-based method calculated by XLOGP program, version 3.2.2, courtesy of CCBG, Shanghai Institute of Organic Chemistry	7.14
Log $P_{o/w}$ (WLOGP)<br>**WLOGP:** Atomistic method implemented from Wildman SA and Crippen GM. 1999 J. Chem. Inf. Model.	6.00
Log $P_{o/w}$ (MLOGP)	5.10

Solubility	2.23e-05 mg/ml ; 5.99e-08 mol/l
Class<br>**Solubility class:** Log *S* scale Insoluble < -10 < Poorly < -6 < Moderately < -4 < Soluble < -2 Very < 0 < Highly	Poorly soluble
Log *S* (SILICOS-IT)<br>**SILICOS-IT:** Fragmental method calculated by FILTER-IT program, version 1.0.2, courtesy of SILICOS-IT, http://www.silicos-it.com	-8.92
Solubility	4.48e-07 mg/ml ; 1.21e-09 mol/l
Class<br>**Solubility class:** Log *S* scale Insoluble < -10 < Poorly < -6 < Moderately < -4 < Soluble < -2 Very < 0 < Highly	Poorly soluble

Pharmacokinetics

GI absorption<br>**Gatrointestinal absorption:** according to the white of the BOILED-Egg	Low
BBB permeant	No

**MLOGP:** Topological method implemented from Moriguchi I. et al. 1992 Chem. Pharm. Bull. Moriguchi I. et al. 1994 Chem. Pharm. Bull. Lipinski PA. et al. 2001 Adv. Drug. Deliv. Rev.	
Log $P_{o/w}$ (SILICOS-IT)	
**SILICOS-IT:** Hybrid fragmental/topological method calculated by FILTER-IT program, version 1.0.2, courtesy of SILICOS-IT, http://www.silicos-it.com	5.99
Consensus Log $P_{o/w}$	
**Consensus Log $P_{o/w}$:** Average of all five predictions	5.77

**BBB permeation:** according to the yolk of the BOILED-Egg	
P-gp substrate	
**P-glycoprotein substrate:** SVM model built on 1033 molecules (training set) and tested on 415 molecules (test set) 10-fold CV: ACC=0.72 / AUC=0.77 External: ACC=0.88 / AUC=0.94	Yes
CYP1A2 inhibitor	
**Cytochrome P450 1A2 inhibitor:** SVM model built on 9145 molecules (training set) and tested on 3000 molecules (test set) 10-fold CV: ACC=0.83 / AUC=0.90 External: ACC=0.84 / AUC=0.91	No
CYP2C19 inhibitor	
**Cytochrome P450 2C19 inhibitor:** SVM model built on 9272 molecules (training set) and tested on 3000 molecules (test set) 10-fold CV: ACC=0.80 / AUC=0.86 External: ACC=0.80 / AUC=0.87	Yes

CYP2C9 inhibitor<br><br>**Cytochrome P450 2C9 inhibitor:** SVM model built on 5940 molecules (training set) and tested on 2075 molecules (test set) 10-fold CV: ACC=0.78 / AUC=0.85 External: ACC=0.71 / AUC=0.81	No
CYP2D6 inhibitor<br><br>**Cytochrome P450 2D6 inhibitor:** SVM model built on 3664 molecules (training set) and tested on 1068 molecules (test set) 10-fold CV: ACC=0.79 / AUC=0.85 External: ACC=0.81 / AUC=0.87	Yes
CYP3A4 inhibitor<br><br>**Cytochrome P450 3A4 inhibitor:** SVM model built on 7518 molecules (training set) and tested on 2579 molecules (test set) 10-fold CV: ACC=0.77 / AUC=0.85 External: ACC=0.78 / AUC=0.86	No
Log K_p (skin permeation)	-3.50 cm/s

**Skin permeation:** QSPR model implemented from Potts RO and Guy RH. 1992 Pharm. Res.	
	Druglikeness
Lipinski	
**Lipinski (Pfizer) filter:** implemented from Lipinski CA. et al. 2001 Adv. Drug Deliv. Rev. MW ≤ 500 MLOGP ≤ 4.15 N or O ≤ 10 NH or OH ≤ 5	Yes; 1 violation: MLOGP>4.15
Ghose	
**Ghose filter:** implemented from Ghose AK. et al. 1999 J. Comb. Chem. 160 ≤ MW ≤ 480 -0.4 ≤ WLOGP ≤ 5.6 40 ≤ MR ≤ 130 20 ≤ atoms ≤ 70	No; 1 violation: WLOGP>5.6
Veber	
**Veber (GSK) filter:** implemented from Veber DF. et al. 2002 J. Med. Chem. Rotatable bonds ≤ 10 TPSA ≤ 140	Yes
Egan	No; 1 violation: WLOGP>5.88
**Egan (Pharmacia) filter:** implemented from	

Egan WJ. et al. 2000 J. Med. Chem. WLOGP ≤ 5.88 TPSA ≤ 131.6	
Muegge	
**Muegge (Bayer) filter:** implemented from Muegge I. et al. 2001 J. Med. Chem. 200 ≤ MW ≤ 600 -2 ≤ XLOGP ≤ 5 TPSA ≤ 150 Num. rings ≤ 7 Num. carbon > 4 Num. heteroatoms > 1 Num. rotatable bonds ≤ 15 H-bond acc. ≤ 10 H-bond don. ≤ 5	No; 1 violation: XLOGP3>5
Bioavailability Score	
**Abbott Bioavailability Score:** Probability of F > 10% in rat implemented from Martin YC. 2005 J. Med. Chem.	0.55

Medicinal Chemistry

PAINS	
**Pan Assay Interference Structures:** implemented from Baell JB. & Holloway GA. 2010 J. Med. Chem.	0 alert
Brenk	1 alert: stilbene

**Structural Alert:** implemented from Brenk R. et al. 2008 ChemMedChem	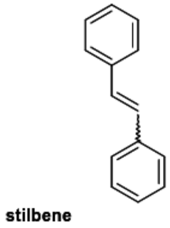
Leadlikeness	
**Leadlikeness:** implemented from Teague SJ. 1999 Angew. Chem. Int. Ed. 250 ≤ MW ≤ 350 XLOGP ≤ 3.5 Num. rotatable bonds ≤ 7	No; 3 violations: MW>350, Rotors>7, XLOGP3>3.5
Synthetic accessibility	
**Synthetic accessibility score:** from 1 (very easy) to 10 (very difficult) based on 1024 fragmental contributions (FP2) modulated by size and complexity penaties, trained on 12’782’590 molecules and tested on 40 external molecules (r^2 = 0.94)	3.01

Swiss Institute of Bioinformatics - © 2025
